# Role of liver enzymes in patients with blunt abdominal trauma to diagnose liver injury

**DOI:** 10.1186/s12245-021-00332-1

**Published:** 2021-01-19

**Authors:** Anup Shrestha, Harish Chandra Neupane, Kishor Kumar Tamrakar, Abhishek Bhattarai, Gaurav Katwal

**Affiliations:** Department of General Surgery, Chitwan Medical College Teaching Hospital, Bharatpur, Nepal

**Keywords:** Blunt abdominal trauma, Liver injury, Liver enzymes, AST, ALT

## Abstract

**Background:**

The liver is the second most injured organ following blunt abdominal trauma (BAT) after the spleen. Although the computed tomography (CT) scan is considered as the gold standard for diagnosing liver injury in BAT, it may not readily available in all the hospitals. This study was performed to evaluate the role of aspartate transaminase (AST) and alanine transaminase (ALT) in patients with BAT and its significance in predicting the diagnosis and severity of the liver injury.

**Method:**

The study was conducted in Chitwan Medical College Teaching Hospital (CMCTH) from February 2019 to May 2020. It was a prospective observational study. All the patients with BAT were received by on-duty surgical residents in the emergency department. Based on the imaging and operative finding, patients with liver injury and without liver injury were noted with the associated injury. For comparisons of clinical and grading characteristics between the two groups (liver injury and no liver injury), the chi-squared test was used for categorical variables as appropriate, and the Mann-Whitney U test used for quantitative variables (AST and ALT). The comparisons between more than two groups (grade of injury) were performed using the Kruskal-Wallis test. The receiver operating characteristic (ROC) was used to calculate the optimal cut-off value of AST and ALT.

**Results:**

Among the 96 patients admitted with BAT, 38 patients had liver injury and 58 patients had no liver injury. The median length of the intensive care unit (ICU) stay of patients with liver injury was higher than without liver injury. There was a significant difference in the median level of AST and ALT (< 0.001) between patients with liver injury and no liver injury. The area under the ROC curve of AST was 0.89 (95% confidence interval 0.86–0.98) and of ALT was 0.92 (95% confidence interval 0.83–0.97). The area under the curve demonstrated that the test was a good predictor for the identification of liver injury and also the severity of liver enzymes. The cut-off values for the liver injury were 106 U/l and 80 U/l for AST and ALT, respectively. Based on these values, AST ≥ 106 U/l had a sensitivity of 71.7%, a specificity of 90%, a positive predictive value of 86.8%, and a negative predictive value of 77.6%. The corresponding values for ALT ≥ 80 U/l were 77.8%, 94.1%, 92.1%, and 82.8%, respectively.

**Conclusion:**

In conclusion, we report the optimal cut-off value of AST and ALT for liver injury in BAT as ≥ 106 U/l and 80 U/l, respectively. The elevated level of AST and ALT might assist the emergency physicians and surgeons to timely refer the suspected patients with the liver injury to a tertiary center.

**Supplementary Information:**

The online version contains supplementary material available at 10.1186/s12245-021-00332-1.

## Introduction

Blunt abdominal trauma (BAT) is one of the most common scenarios in the emergency department (ED). The prevalence of intra-abdominal injury in patients with blunt abdominal trauma among is 13% [1]. Motor vehicle accident is one of the major causes of BAT. Other causes include fall injury, physical assault, sports, and crush injury [2]. The liver is the second most injured organ following BAT after the spleen [3]. The clinical diagnosis of liver injury in BAT is a major challenge for emergency physicians and trauma surgeons.

Focus Assessment with Sonography for Trauma (FAST) scan is one of the useful radiological investigations of BAT but has low sensitivity in diagnosing the liver injury and is user-dependent. Therefore, computed tomography (CT) scan is considered as the gold standard for diagnosing liver injury in BAT [4, 5]. CT scan will help to access not only the liver but also other associated organ injuries. Not all the health facilities will have access to the CT scan. In these centers, the elevation of liver enzymes, i.e., aspartate transaminase (AST) and alanine transaminase (ALT), may provide valuable guidance to the emergency physician to suspect liver trauma. Also, the CT scan is expensive and has exposure hazards. It is also difficult to maintain the resuscitation of the hemodynamically unstable patient in the CT scan suite. This might be an extra burden for patients not only in developing countries like Nepal but also for the health system in a developed country. Previous studies have shown that these parameters may assist in the prediction of liver trauma and their severity [6–10]. Patients will be greatly benefitted from on timely referral of the patient to the tertiary trauma center.

So, the objective of this study was to evaluate the role of AST and ALT in patients with BAT and its significance in predicting the severity of the liver injury.

## Methods and material

The study was conducted in Chitwan Medical College Teaching Hospital (CMCTH) which was established in 2006. Since then, CMCTH has developed as a multi-specialty tertiary care center in Nepal. The ED receives a huge number of trauma casualties from all over central Nepal. It was a prospective observational study from February 2019 to May 2020.

### Inclusion criteria

- All the patients with blunt abdominal trauma who were admitted at CMCTH.

### Exclusion criteria

- Patients with penetrating abdominal trauma
- Patients who died in the emergency department during resuscitation
- Patients who presented late after 24 h of the trauma
- Patients with a history of liver disease
- Patients positive for hepatitis B and hepatitis C surface antigen

### Study method

All the patients with BAT were received by on-duty surgical residents in the emergency department. The patient was initially evaluated in the triage, and necessary resuscitation was done according to the Advanced Trauma Life Support (ATLS) protocol. Then, blood samples were sent for hemoglobin, hematocrit, WBC (white blood cell) count, serum AST, and ALT. FAST scan was done and patients with hemodynamic instability were taken for laparotomy. CT scans were done in the rest of the stable patients. The on-duty surgical residents inform the attending surgeon on duty. The first author or the attending surgeon further evaluated the patient and appropriate history with age, gender, mode of injury, and time of trauma of the patient were recorded. Based on the imaging and operative finding, patients with liver injury and without liver injury were noted with the associated injury. The grade of liver injury was done according to the Organ injury Scale by the American Association of Surgery for Trauma (AAST) (2018 version) (Additional file 1: Supplementary Table) [11]. The datasheet was completed by the first author on the same day of admission or within 24 h of the admission. All the patients with BAT were included in the study as they are received and managed by the surgeons of the Department of Surgery of CMCTH. On discharge, the length of total hospital stay, length ICU stay (if admitted in ICU), re-admission in ICU, any blood transfusion, morbidity, and mortality were also recorded.

### Data analysis

All statistical analyses and graphics were performed with the SPSS for Windows, version 16.0., Chicago, SPSS Inc. For comparisons of clinical and grading characteristics between the two groups (liver injury and no liver injury), the chi-squared test was used for categorical variables as appropriate, and the Mann-Whitney *U* test used for quantitative variables (AST and ALT). The comparisons between more than two groups (grade of injury) were performed using the Kruskal-Wallis test. Results were expressed as median (IQR). All *p* values are two-sided with *p* values < 0.05 considered statistically significant. The receiver operating characteristic (ROC) was used to calculate the optimal cut-off value of AST and ALT, and using the optimal cut-off value, the sensitivity, specificity, positive predictive value, and negative predictive value were obtained [12].

### Ethics statement

The institutional review committee of CMCTH approved this prospective observational study. Written consent was given by patients for the information to be used for the research.

## Results

### Patient’s demographics

Among the 96 patients admitted with BAT, 38 patients had liver injury and 58 patients had no liver injury. The quantitative data were analyzed in the median because of the skewed distribution. The median age for liver injury was 27 years old. 78.9% of patients with liver injury were male, and motor vehicle accident was the most common mode of injury with 71.1%. Five patients with liver injury had a negative FAST scan. Eight patients with hemodynamic instability were taken directly to the operation room for emergency laparotomy, and the rest of the patients underwent a CT scan. Only 1 patient with liver injury was found during emergency laparotomy. The demographic profile of patients divided into two groups with liver injury and no liver injury is given in Table 1. There were no significant differences in ICU admission rate and days of hospital or ICU stay between the liver injury and no liver injury groups, whereas there was a significant difference in the mortality rate (*p* < 0.05).

**Table 1:** Demographic features of liver injury and non-liver injury patients

	Liver injury (*n* = 38)	Non-liver injury (*n* = 58)	*p* value
Age, median (range) years	27.0 (3–76)	31.50 (6–83)	0.848
Gender, *n* (%)			0.236
Male	30 (78.9)	51 (87.9)	
Female	8 (21.1)	7 (12.1)	
Mechanism, *n* (%)			0.654
Motor vehicle accident	27 (71.1)	42 (72.4)	
Fall from height	7 (18.4)	12 (20.7)	
Physical assault	4 (10.5)	3 (5.2)	
Crush injury	0	1 (1.7)	
CT scan, *n* (%)			0.102
Performed	37 (97.4)	51 (87.9)	
Not performed	1 (2.6)	7 (12.1)	
ICU admission, *n* (%)			0.338
Admitted	35 (92.1)	56 (96.6)	
Not admitted	3 (7.9)	2 (3.4)	
Re-admission in ICU, *n* (%)			0.854
Yes	3 (7.9)	4 (6.9)	
No	35 (92.1)	54 (93.1)	
Surgical intervention (all surgery), *n* (%)			0.494
Performed	15 (39.5)	27 (46.6)	
Not performed	23 (60.5)	31 (53.4)	
ICU stay, median, (range) days	3 (0–22)	3 (0–26)	0.721
Total hospital, median (range) days	9 (2–42)	9 (1–60)	0.997
Mortality, *n* (%)			0.536
Yes	2 (5.3)	5 (8.6)	
No	36 (94.7)	53 (91.4)	

### Liver injuries

There were 4 (10.5%) patients with grade I injuries and 11 (28.9%) with grade II injuries, 18 (47.4%) with grade III injuries, and 5 (13.2%) with grade IV injuries. There was no grade V injury reported in our study. Out of 38 patients with liver injury, only 8 (21.1%) patients had isolated liver injury while 30 (78.9%) patients had a liver injury with combined injuries following BAT. Of the 30 patients with combined injuries with liver injury, 18 had a chest injury, 24 had other abdominal injuries, 9 had head plus spine injury, and 10 had pelvis plus extremities injury. The most common associated organ injured with liver injury was the spleen with 37.5%. The patient with a liver injury with combined other associated injury tended to stay at ICU more days than with isolated liver injuries. Thirty-three (86.8%) patients out of 38 patients were treated conservatively. There was a significant difference in the median of hemoglobin (*p* < 0.05), hematocrit (*p* < 0.05), AST (*p* < 0.001), and ALT (*p* < 0.001) between different grades of liver injury (Table 2), while there was no difference in total ICU stay and hospital stay. The median of laboratory parameters and total ICU and hospital stay of each grade of liver injury is given in Table 3.

**Table 2:** Laboratory parameters of patients with liver injury and no liver injury

Laboratory parameters	Patients with liver injury	Patients with no liver injury	*p* value
WBC, median (range) mm^−3^	10450 (4650–20800)	10000 (1990–25010)	0.943
Hb% median (range) g/dl	11.4 (6.8–15.6)	12.8 (5.6–17.2)	0.067
PCV median (range) %	34.5 (21.20–45.50)	36.35 (17.40–48)	0.232
AST median (range) U/l	379 (26–6080)	46 (17–339)	< 0.001
ALT median (range) U/l	290.5 (27–2681)	39 (12–415)	< 0.001

**Table 3:** Laboratory parameters and outcome of each grade of liver injury (the Kruskal-Wallis test)

	Grade I (*n* = 4)	Grade II (*n* = 11)	Grade III (*n* = 18)	Grade IV (*n* = 5)	*p* value
WBC, median (range) mm^−3^	12,945 (10,300–14,480)	8990 (4650–20,800)	8700 (4900–14,900)	12,150 (6900–18,800)	0.541
Hb%, median (range) g/dl	14.1 (6.8–15.6)	11.4 (9.0–14.8)	11.55 (7.5–14.8)	9 (7.7–9.9)	0.028*
PCV, median (range) %	40.75 (23–45.30)	39.10 (28.60–45)	34.80 (21.30–44.10)	23.50 (21.20–29.60)	0.027*
AST, median (range) U/l	173 (26–317)	134 (47–888)	341 (38–6080)	619 (432–1800)	< 0.001
ALT, median (range) U/l	144 (27–332)	118 (33–787)	263 (48–2681)	559 (324–2300)	< 0.001
Total hospital stay, median (range) days	6.5 (4–10)	9 (4–30)	8 (2–25)	11 (5–40)	0.662
Total ICU stay, median (range) days	1 (0–3)	2 (0–22)	2.5 (1–13)	5 (3–10)	0.161

*p < 0.05

### Main results

**Fig. 1:**
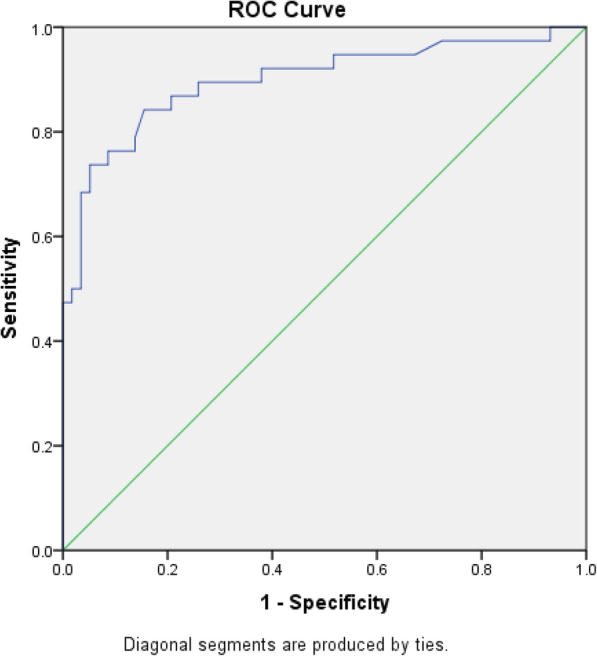
ROC curve of AST. ROC receiver operating characteristic, AST aspartate aminotransferase

**Fig. 2:**
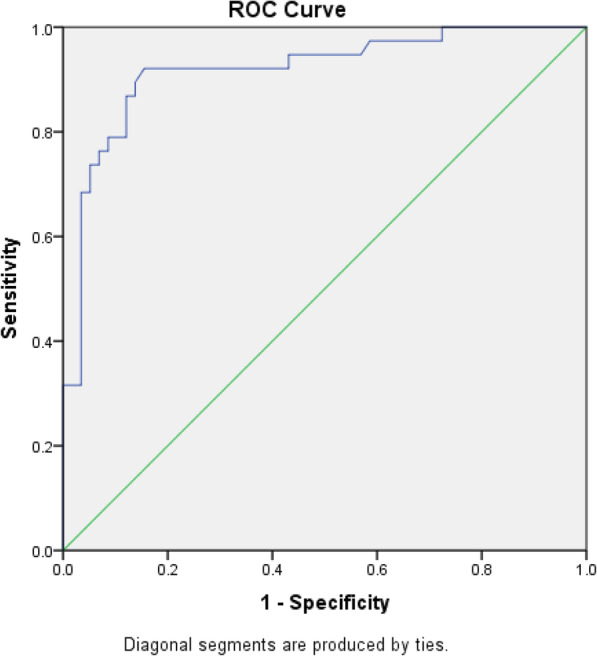
ROC curve of ALT. ROC receiver operating characteristic, ALT alanine aminotransferase

The clinical outcome of the patients with liver trauma is given in Table 4. The statical significance in AST and ALT level was seen only in the mortality rate of the patients whereas no significant difference was seen in ICU admission, surgical intervention for liver injury, and blood transfusion category. The receiver operating characteristic (ROC) curve analysis for AST and ALT is showed in Figs. 1 and 2, respectively. The area under the ROC curve of AST was 0.89 (95% confidence interval 0.86–0.98) and of ALT was 0.92 (95% confidence interval 0.83–0.97). The area under the curve demonstrated that the test was a good predictor for the identification of liver injury. According to the ROC curve, the cut-off values for the liver injury were 106 U/l and 80 U/l for AST and ALT, respectively. Using the cut-off value, sensitivity, specificity, positive predictive value, and negative predictive value were calculated (Table 5).

**Table 4:** Clinical outcome of the patients with liver injury and their AST and ALT levels

Outcome	*n* (%)	Median AST (range) U/l	*p* value	Median ALT (range) U/l	*p* value
ICU admission			0.685		0.978
Yes	35 (92.1)	323 (38–6080)		256 (33–2681)	
No	3 (7.9)	317 (26–604)		332 (27–638)	
Surgical intervention (liver injury related)			0.110		0.088
Yes	5 (13.2)	619 (323–1800)		569 (324–2300)	
No	33 (86.8)	314 (26–6080)		241 (27–2681)	
Mortality			0.022		0.019*
Yes	2(5.3)	3940 (1800–6080)		2490.5 (2300–2681)	
No	36(94.7)	314 (26–1993)		248.5 (27–787)	
Blood transfusion done			0.381		0.154*
Yes	9 (23.7)	359 (47–6080)		569 (93–2681)	
No	29 (76.3)	314 (26–1993)		241 (27–667)	

*p < 0.05

**Table 5:** Sensitivity and specificity of AST and ALT for liver injury

	AST ≥ 106 U/l (%)	ALT ≥ 80 U/l (%)
Sensitivity	71.7	77.8
Specificity	90	94.1
Positive predictive value	86.8	92.1
Negative predictive value	77.6	82.8

## Discussion

Before CT scans were introduced, surgeons use to proceed for laparotomy when they suspected parenchymatous organ injury in BAT [13]. The availability of a modern multi-detector CT scan has helped today’s surgeons tremendously in managing BAT with liver injury conservatively. It not only helps to establish the grade of injury but also helps to detect delayed complications of the high-grade liver in injury [14, 15]. The diagnosis of liver injury is challenging in peripheral centers all over the world where CT is not available. This even applies to developed countries like Japan. To establish the severity of the liver injury is beyond the reach of those emergency physicians and surgeons.

The development of the FAST scan is useful in diagnosing hemoperitoneum, but because of its low sensitivity and specificity, its role in BAT is limited [4, 16]. The role of elevated liver enzymes in predicting the severity of liver injuries is still a matter of dispute. Liver enzymes AST and ALT are present in hepatocytes in high concentration, and following BAT, they leak into blood circulation. Their main function is to catabolize amino acids, permitting them to enter the citric acid cycle. AST is typically found in the liver only but ALT is also found in the heart skeletal muscle, kidney, brain, and RBC [17]. The alteration of ALT and AST in chronic liver injury and drug-induced liver has been extensively studied [18]. Few studies have demonstrated their role as a marker in predicting the severity of liver injury [6–10].

In this prospective observational study, we investigate the role of AST and ALT in the diagnosis of liver injury and its severity. In the recent study, Koyoma et. al. reported the optimal cut-off value of AST and ALT was 109 U/l and 97 U/l respectively for the patients with liver injury in blunt abdominal trauma. They suggested the optimal cut-off value as a predictor and also screening tool for CT scans for the presence of liver injury. Even in a developed country like Japan, they have pointed out the significance of AST and ALT levels for early CT scans and if not available, transfer to the patient to tertiary center [10]. Similar results were reported by Tian et al., Chang et al., Shrivastava et al., and Lee et al. [6–9]. Shrivastava et al. only compared ALT level whereas other studies included both AST and ALT values.

Our study results also demonstrate that the increased level of AST and ALT predicts the underlying liver injury in patients with blunt abdominal trauma. The median level of grade II liver injury was less than grade I liver injury. This may be because of very few patients with grade I liver injury. The median level of grade III and grade IV was much higher than grades I and II (Table 3). In countries like Nepal where there are few tertiary centers, patients with a high level of AST and ALT should be stabilized and immediately shifted to tertiary care centers. The median of AST and ALT of patients requiring blood transfusion was more than that of the patient not requiring blood transfusion (*p* < 0.05). This shows that the AST and ALT level is not only important for the prediction of liver injury but also alerts the surgeons about the possible need for blood transfusion. Similarly, the median of AST and ALT in patients with mortality was significantly higher than patients without mortality (*p* < 0.05). Since only 2 patients expired due to liver injury, the significance of AST and ALT in mortality cannot be suggested. One study reported elevated WBC counts together with elevated AST and ALT are strongly associated with liver injury [19]. But in our study, there was no association between WBC count and liver injury. Overall, the sensitivity, specificity, and positive and negative predictive value of AST and ALT for predicting liver injury were low, so we suggest not using these liver enzymes as a diagnostic tool but to use as a screening tool for possible liver injury. There were some limitations during the study. This was a single institute study and the number of patients with liver injury was relatively small particularly grade I liver injury.

## Conclusion

In conclusion, we report the optimal cut-off value of AST and ALT for liver injury in BAT as ≥ 106 U/l and 80 U/l, respectively. In countries like Nepal, where CT scan is not available in every center, the elevated level of AST and ALT might assist the surgeons to timely refer the suspected patients with the liver injury to a tertiary center. In tertiary centers, it might help the surgeons to go for conservative management for minor liver injuries in BAT preventing the exposure hazards of CT scans.

## Supplementary Information

**Additional file 1.** Supplementary Table.

## Data Availability

The data that support the findings of this study are available from the corresponding author upon reasonable request.

## Abbreviations

BAT: Blunt abdominal trauma
CT: Computed tomography
AST: Aspartate transaminase
ALT: Alanine transaminase
ED: Emergency department
CMCTH: Chitwan Medical College Teaching Hospital
ATLS: Advanced Trauma Life Support
GCS: Glasgow coma scale
WBC: White blood cell
ICU: Intensive care unit
ROC: Receiver operating characteristic
