# Supplementary material for: Role of liver enzymes in patients with blunt abdominal trauma to diagnose liver injury
Source: Int J Emerg Med. 2021 Jan 19;14:7. doi: 10.1186/s12245-021-00332-1 (PMC7814543; doi:10.1186/s12245-021-00332-1)
Supplement: Supplementary file 1 — Additional file 1. Supplementary Table. [file 12245_2021_332_MOESM1_ESM.docx]

**Supplementary Table: Liver injury Scale by AAST (American Association for the Surgery of Trauma) 2018**

| **AAST Grade** | **Imaging Criteria (CT finding)** | **Operative Finding** |
| --- | --- | --- |
| I | – Subcapsular hematoma <10% surface area  – Parenchymal laceration <1 cm in depth | – Subcapsular hematoma <10% surface area  – Parenchymal laceration <1 cm in depth  Capsular tear |
| II | – Subcapsular hematoma 10–50% surface area; intraparenchymal hematoma <10 cm in diameter  – Laceration 1–3 cmin depth and ≤ 10 cm length | – Subcapsular hematoma 10–50% surface area; intraparenchymal hematoma <10 cm in diameter  – Laceration 1–3 cm in depth and ≤ 10 cm length |
| III | – Subcapsular hematoma >50% surface area; ruptured subcapsular or parenchymal hematoma  – Intraparenchymal hematoma >10 cm  – Laceration >3 cm depth  – Any injury in the presence of a liver vascular injury or active bleeding contained within liver parenchyma | – Subcapsular hematoma >50% surface area or expanding; ruptured subcapsular or parenchymal hematoma  – Intraparenchymal hematoma >10 cm  – Laceration >3 cm in depth |
| IV | – Parenchymal disruption involving 25–75% of a hepatic lobe  – Active bleeding extending beyond the liver parenchyma into the peritoneum | – Parenchymal disruption involving 25–75% of a hepatic lobe |
| V | – Parenchymal disruption >75% of hepatic lobe  –Juxtahepatic venous injury to include retrohepatic vena cava and central major hepatic veins | – Parenchymal disruption >75% of hepatic lobe  – Juxtahepatic venous injury to include retrohepatic vena cava and central major hepatic veins |
